# Point-of-care testing for procalcitonin in identifying bacterial infections in young infants: a diagnostic accuracy study

**DOI:** 10.1186/s12887-018-1349-7

**Published:** 2018-12-12

**Authors:** Thomas Waterfield, Julie-Ann Maney, Martin Hanna, Derek Fairley, Michael D. Shields

**Affiliations:** 1 Centre for Experimental Medicine, Wellcome Wolfson Institute of Experimental Medicine, Queen’s University Belfast, 97 Lisburn Road, Belfast, BT9 7AE UK; 2 Belfast Health & Social Care Trust, Belfast, UK

**Keywords:** Pediatrics, Infection, Sepsis, Biomarker, Procalcitonin, PCT, Infant, Febrile

## Abstract

**Background:**

The primary objective of this study was to report on the diagnostic accuracy of point-of-care testing (POCT) for procalcitonin (PCT) in identifying invasive bacterial infections in young infants. Invasive bacterial infection was defined as the isolation of a bacterial pathogen in blood or cerebrospinal fluid culture.

**Methods:**

This was a prospective observational diagnostic accuracy study. Young infants less than 90 days of age presenting to the Royal Belfast Hospital for Sick Children with signs of possible bacterial infection were eligible for inclusion. Eligible infants underwent point-of-care testing for procalcitonin in the emergency department. Testing was performed by clinical staff using 0.5 ml of whole blood. Results were available within 20 min.

**Results:**

126 children were included over a 5-month period between September 2017 and January 2018. There were 14 children diagnosed with bacterial infections (11.1%). Of these 4 children were diagnosed with invasive bacterial infections (3.2%). POCT procalcitonin demonstrated an excellent diagnostic accuracy for identifying children with invasive bacterial infection area under the curve (AUC) of 0.97(95% CI, 0.94 to 1.0). At a cut-off value of 1.0 ng/ml is highly accurate at identifying infants at risk of invasive bacterial infection with a sensitivity and specificity of 1.00 and 0.92 respectively.

**Conclusions:**

Point-of-care procalcitonin can be performed quickly in the emergency department and demonstrates an excellent diagnostic accuracy for the identification of young infants with invasive bacterial infections.

**Trial registration:**

NCT03509727 Retrospectively registered on 26th April 2018.

## Background

Young febrile infants under 3 months of age are typically treated as a high-risk group for invasive bacterial infection with many receiving parenteral antibiotics [1]. Over the last 20 years a number of attempts have been made to identify those young infants in whom the risk of invasive bacterial infection is low so that antibiotics could be safely withheld. These include the Rochester, Philadelphia, Boston criteria and more recently the Step-By-Step approach [2–5].

In the UK the current standard of practice for the management of febrile children comes from the National Institute for Health and Care Excellence (NICE) and the clinical practice guideline 160 “Fever in under 5s: assessment and initial management” [1]. In that guidance young febrile infants less than 3 months of age are identified as high risk of serious bacterial infection; with the advice that all febrile infants under 1 month of age and any “unwell” appearing febrile young infants are administered immediate parenteral antibiotics [1]. The NICE guidance appropriately advocates a low threshold for parental antibiotics with the acceptance that some children will be treated unnecessarily. This approach reflects the challenge of recognising early serious bacterial infection in this group. This necessarily cautious approach needs balanced with the need for better anti-microbial stewardship to prevent the spread of multi-drug resistant bacteria [6, 7].

There is therefore a need for improved diagnostics to help better identify those infants requiring immediate parental antibiotics from those who do not. This has led to a growing interest in point-of-care testing (POCT) for biomarkers of infection including procalcitonin (PCT) [5, 8–14]. In the UK procalcitonin is not routinely used in the assessment of young febrile infants as it is not currently recommended by NICE. NICE have however, called for additional research into the diagnostic accuracy of PCT in the assessment of febrile children [1].

At the Royal Belfast Hospital for Sick Children we introduced point-of-care testing for PCT to specifically help identify invasive bacterial infections in young infants.

### Objectives

The primary objective of this study was to assess the diagnostic accuracy of point-of-care testing for PCT in identifying young infants with invasive bacterial infections.

The secondary objective was to determine the diagnostic accuracy of point-of-care testing for PCT in identifying young infants with any bacterial infection (invasive or non-invasive).

## Methods

### Study design

The RBHSC is Northern Ireland’s only tertiary paediatric children’s hospital and has 40,000 attendances to the emergency department annually. This prospective diagnostic accuracy study was conducted from the 1st of September 2017 until the 31st January 2018.

Case report forms were prospectively completed for all those undergoing PCT testing. The study was designed to adhere to the STARD criteria for reporting diagnostic accuracy studies [15].

### Data collection

Anonymised data were collected on standardised case report forms. Data collected included age, gender, temperature on arrival, PCT result and final diagnosis including culture results and molecular testing.

### Inclusion criteria

Any child under 90 days of age presenting with signs or symptoms suggestive of possible bacterial infection. The NICE guidance CG160 “Fever in under 5s: assessment and initial management” was used as a guide for clinicians (Table 1). Children were included at the discretion of the attending clinician.

**Table 1:** Signs and symptoms of serious illness as defined by NICE CG160 – “Fever in under 5s: assessment and initial” [1]

High Risk Features	Intermediate Risk Features
Pale/mottled/ashen/blue skin, lips or tongue	Pallor of skin, lips or tongue reported by parent or carer
No response to social cues	Not responding normally to social cues
Appearing ill to a healthcare professional	No smile
Does not wake or if roused does not stay awake	Wakes only with prolonged stimulation
Weak, high-pitched or continuous cry	Decreased activity
Grunting	Nasal flaring
Respiratory rate greater than 60 breaths per minute	Dry mucous membranes
Moderate or severe chest indrawing	Poor feeding in infants
Reduced skin turgor	Reduced urine output
Bulging fontanelle.	Rigors

### Exclusion criteria

There were no exclusion criteria for this study. Any child under 90 days of age (uncorrected for gestational age) with signs of infection as outlined by NICE guidance “Fever in under 5s: assessment and initial management” was eligible for inclusion (Table 1).

The broad inclusion and exclusion criteria were chosen to (i) best reflect current clinical practice in the UK (ii) to minimise selection bias and (iii) provide clinically applicable results for clinicians.

### Index test

Procalcitonin was tested using the commercially available, CE marked, BRAHMS procalcitonin assay on the Samsung LABGEO IB10® analyser in the paediatric emergency department. All testing was performed by emergency department staff using 0.5 ml of whole blood and as per the manufacturer’s instructions. Testing was performed immediately after collecting the blood sample and results were available within 20 min. In all instances the procalcitonin result was available before the reference standard.

### Reference standards

Invasive bacterial infection (IBI) defined as isolation of a bacterial pathogen in blood or cerebrospinal fluid culture. *Staphylococcus epidermidis* and *Streptococcus viridans* were considered contaminants.

Non-invasive bacterial infections (Non-IBI) defined as:

- Urinary Tract Infection (UTI) – Growth of > 10,0000 cfu/ml of a single organism from either a single invasive sample (catheter or suprapubic aspiration) or two non-invasive samples.
- Bacterial gastroenteritis - Isolation of bacteria in stool.
- Pneumonia - Focal consolidation on chest radiograph confirmed by an experienced paediatric radiologist.

In all instances the technicians performing the reference standard testing were blinded to the results of the index tests.

### Statistical analysis

Normally distributed data were expressed as mean (Standard Deviation, SD), non-normally distributed data as median and interquartile range, and categorical variables were reported as percentages. We calculated the sensitivity and specificity of procalcitonin at a range of pre-determined cut-offs. The area under the receiver operating characteristic (ROC) curves for procalcitonin were reported for both invasive and all bacterial infections. Analysis was performed using IBM SPSS Statistics Version 23.

### Ethical approval

Procalcitonin testing was being introduced as a service development to improve patient care at the RBHSC. This service development project was reviewed and approved by the Belfast Health and Social Care Trust Research and Development office and no Office for Research Ethics Committees approval was deemed necessary.

### Study registration

This study was retrospectively registered at https://www.clinicaltrials.gov (trial registration: NCT03509727) on the 26th April 2018.

## Results

**Fig. 1:**
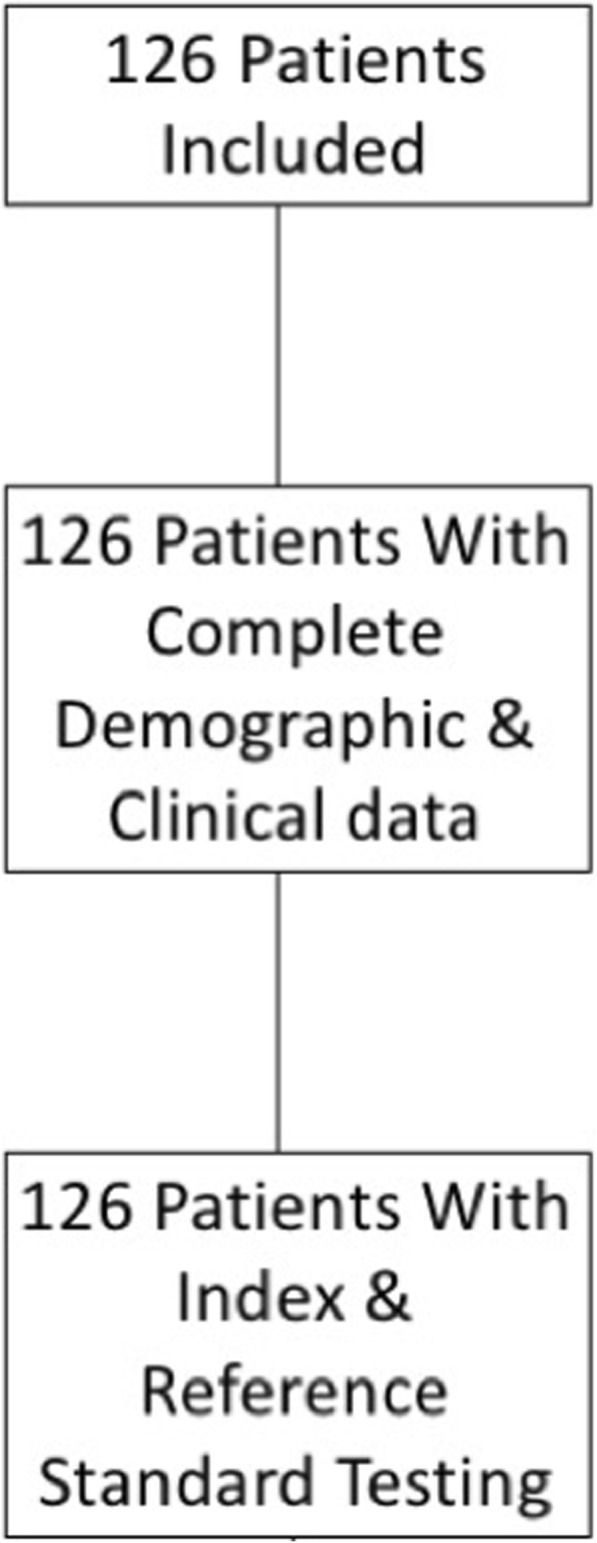
Flow Diagram of Study

A total of 126 children were recruited to the study including 4 children with IBI (all *E. coli* bacteraemia) and 10 children with Non-IBI (all urinary tract infections). All children had complete demographic and clinical data sets. The median age at presentation was 42 days and the median temperature at presentation was 37.4 °C. A summary of the demographic and clinical data is shown in Table 2. A patient flow of results is shown in Fig. 1.

**Table 2:** Epidemiological & Clinical Data of Patients

Epidemiological & Clinical Data	
Male (%)	51
Female (%)	49
Age (Median and interquartile range), days	42 (14–70)
Median Temperature at Presentation (Median and interquartile range), °C	37.4 (35.9–38.9)
Parenteral Antibiotics, %	27.8
Invasive Bacterial Infection, % (All *E. coli* Bacteraemia)	3.2
Non-invasive Bacterial Infection, % (All Urinary Tract Infections)	7.9%

### Diagnostic accuracy of procalcitonin testing for bacterial infections

**Fig. 2:**
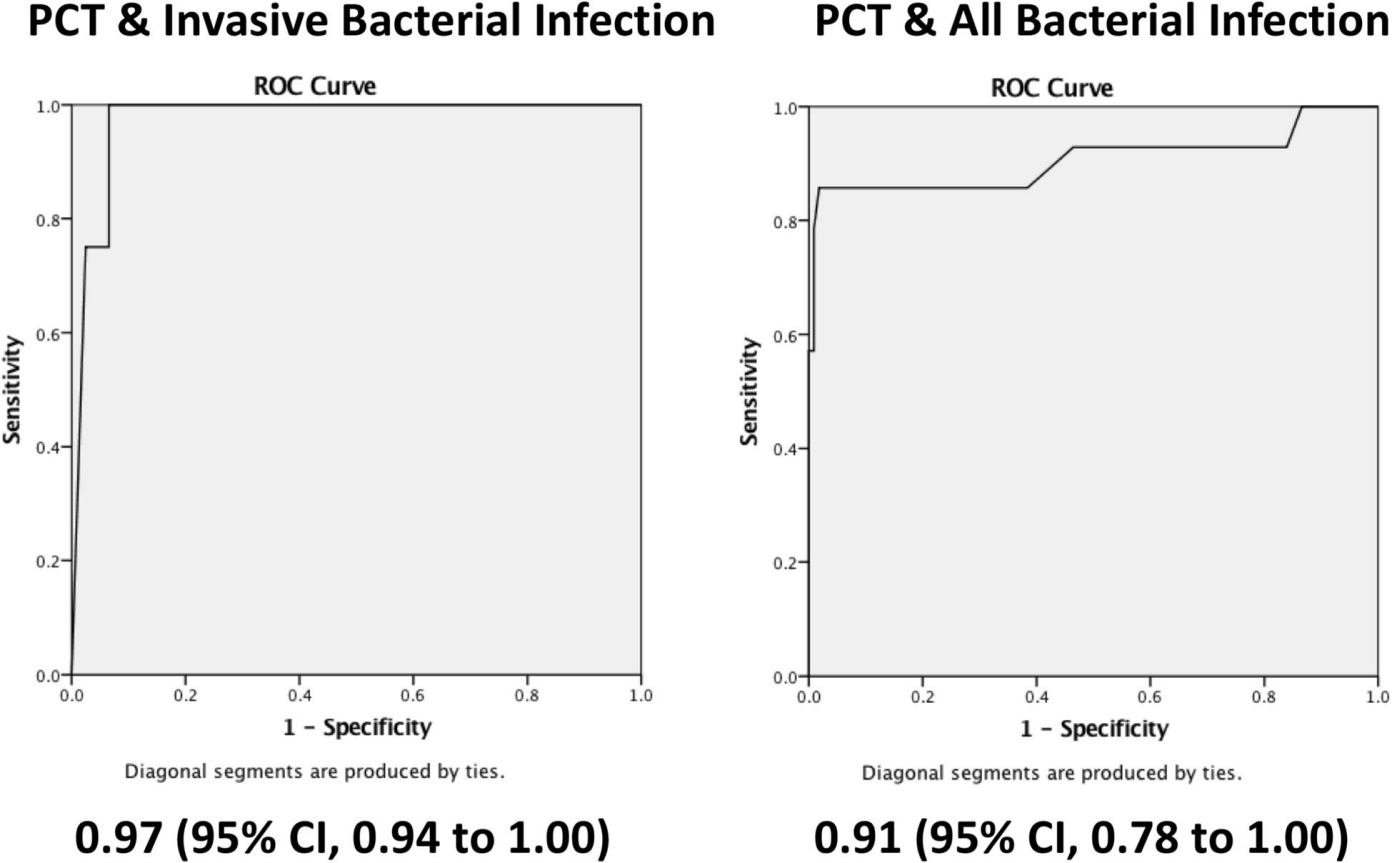
Receiver Operating Characteristic (ROC) Curves for Procalcitonin (PCT)

The area under the curve (AUC) for the receiver operating characteristic (ROC) curve for identifying infants with IBI was 0.97(95% CI, 0.94 to 1.00) and the AUC for identifying infants with all bacterial infections was 0.91(95% CI, 0.78 to 1.00) as shown in Fig. 2. The sensitivity, specificity, negative predictive value (NPV) and positive predictive value of PCT testing over a range of cut-offs is shown in Table 3.

**Table 3:** Diagnostic Accuracy of PCT point-of-care in Identifying Infants with Bacterial Infections with 95% Confidence Intervals

	PCT Cut-off 0.25 ng/ml		PCT Cut-off 0.5 ng/ml		PCT Cut-off 1.0 ng/ml	
	Invasive Bacterial Infection	All Bacterial Infection	Invasive Bacterial Infection	All Bacterial Infection	Invasive Bacterial Infection	All Bacterial Infection
Sensitivity	1.00 (0.40 to 1.00)	0.86 (0.56 to 0.97)	1.00 (0.40 to 1.00)	0.86 (0.56 to 0.97)	1.00 (0.40 to 1.00)	0.86 (0.56 to 0.97)
Specificity	0.72 (0.63 to 0.80)	0.77 (0.68 to 0.84)	0.91 (0.84 to 0.95)	0.97 (0.92 to 0.99)	0.92 (0.85 to 0.96)	0.98 (0.93 to 0.99)
Positive Predictive Value	0.11 (0.03 to 0.26)	0.32 (0.18 to 0.49)	0.27 (0.09 to 0.55)	0.80 (0.51 to 0.95)	0.29 (0.10 to 0.58)	0.86 (0.56 to 0.97)
Negative Predictive Value	1.00 (0.95 to 1.00)	0.98 (0.93 to 1.00)	1.00 (0.96 to 1.00)	0.98 (0.93 to 1.00)	1.00 (0.96 to 1.00)	0.98 (0.93 to 1.00)

### Comparison with laboratory CRP testing

Of the 126 patients that underwent PCT testing 121 also had C-Reactive Protein (CRP) testing performed in the hospital laboratory. This cohort included all of the children with bacterial infections (*n* = 14). In this cohort CRP demonstrated an AUC for identifying infants with IBI of 0.98(95% CI, 0.96 to 1.00) and an AUC for identifying infants with all bacterial infections of 0.98(95% CI, 0.96 to 1.00). The sensitivity, specificity, negative predictive value (NPV) and positive predictive value of PCT testing over a range of cut-offs is shown in Table 4.

**Table 4:** Diagnostic Accuracy of Laboratory CRP in Identifying Infants with Bacterial Infections with 95% Confidence Intervals

	CRP cut-off 20 mg/l		CRP cut-off 50 mg/l		CRP cut-off 100 mg/l	
	Invasive Bacterial Infection	All Bacterial Infection	Invasive Bacterial Infection	All Bacterial Infection	Invasive Bacterial Infection	All Bacterial Infection
Sensitivity	1.00 (0.40 to 1.00)	0.93 (0.64 to 1.00)	1.00 (0.40 to 1.00)	0.62 (0.32 to 0.85)	0.50 (0.01 to 0.91)	0.21 (0.07 to 0.19)
Specificity	0.86 (0.78 to 0.92)	0.94 (0.87 to 0.97)	0.96 (0.89 to 0.98)	0.99 (0.94 to 1.00)	0.99 (0.95 to 1.00)	1.00 (0.96 to 1.00)
Positive Predictive Value	0.20 (0.07 to 0.44)	0.65 (0.41 to 0.84)	0.44 (0.15 to 0.77)	0.89 (0.51 to 0.99)	0.67 (0.13 to 0.98)	1.00 (0.31 to 1.00)
Negative Predictive Value	1.00 (0.95 to 1.00)	0.99 (0.94 to 1.00)	1.00 (0.96 to 1.00)	0.96 (0.9 to 1.00)	0.98 (0.93 to 1.00)	0.91 (0.83 to 0.95)

## Discussion

### Main findings

This study demonstrates that POCT for PCT is highly accurate at identifying infants with invasive bacterial infections AUC 0.97. At a cut-off value of 1.0 ng/ml is highly accurate at identifying infants at risk of invasive bacterial infection with a sensitivity and specificity of 1.00 and 0.92 respectively.

In this study it was not possible to directly compare CRP and PCT due to (i) not all children had both tests performed and (ii) PCT was performed as a rapid point-of-care test whereas CRP was performed in the laboratory. The available data regarding the diagnostic accuracy of CRP in this study does however, suggest that if a rapid and reliable point-of-care CRP test was available it could be expected to perform similarly to that of point-of-care PCT in this population.

### What this adds

Current NICE guidance does not recommend the use of PCT in the assessment of febrile young infants [1]. This is despite large scale multicentre research from across Europe demonstrating the value of PCT testing in this group [5, 14, 16, 17]. NICE state that “the benefit of adding PCT to this schedule is not clear”. This study, although small, demonstrates that POCT procalcitonin testing demonstrates a high diagnostic accuracy for identifying young infants at risk of bacterial infection in a UK population.

The addition of POCT procalcitonin testing to the initial assessment of young infants with signs of infection may improve the early recognition of serious bacterial infection in this group.

### Study limitations and future research

This study is limited in that it was performed over a short duration in a single centre with small numbers of infants. Furthermore, infants were included at the discretion of the attending clinician thereby introducing potential selection bias. This was unavoidable as PCT testing was introduced as service development and this study performed as an observation of routine care. Despite this the rate of bacterial infection diagnosed in this study (11.1%) is slightly lower than reports in other larger studies in Europe and The USA (11.9–23.1%) suggesting that selection bias towards sicker children with greater risk of bacterial infection was unlikely [5, 14, 17–20].

To fully elucidate the value of PCT testing and the most appropriate cut-off value further multicentre prospective studies are required.

## Conclusions

Our data suggests that POCT for procalcitonin demonstrates an excellent diagnostic accuracy for identifying young infants with invasive bacterial infections.

## Abbreviations

AUC: Area Under Curve
NICE: National Institute of Health and Care Excellence
NPV: Negative Predictive Value
PCT: Procalcitonin
POCT: Point-of-care-testing
PPV: Positive Predictive Value
RBHSC: Royal Belfast Hospital for Sick Children
